# Identification of WOX Family Genes in *Selaginella kraussiana* for Studies on Stem Cells and Regeneration in Lycophytes

**DOI:** 10.3389/fpls.2016.00093

**Published:** 2016-02-05

**Authors:** Yachao Ge, Jie Liu, Minhuan Zeng, Jianfeng He, Peng Qin, Hai Huang, Lin Xu

**Affiliations:** ^1^National Laboratory of Plant Molecular Genetics, CAS Center for Excellence in Molecular Plant Sciences, Institute of Plant Physiology and Ecology, Shanghai Institutes for Biological Sciences, Chinese Academy of SciencesShanghai, China; ^2^Department of Instrumentation Science and Engineering, Shanghai Jiao Tong UniversityShanghai, China

**Keywords:** *Selaginella kraussiana*, lycophyte, stem cell, *WOX*, regeneration, vascular plants

## Abstract

Plant stem cells give rise to all tissues and organs and also serve as the source for plant regeneration. The organization of plant stem cells has undergone a progressive change from simple to complex during the evolution of vascular plants. Most studies on plant stem cells have focused on model angiosperms, the most recently diverged branch of vascular plants. However, our knowledge of stem cell function in other vascular plants is limited. Lycophytes and euphyllophytes (ferns, gymnosperms, and angiosperms) are two existing branches of vascular plants that separated more than 400 million years ago. Lycophytes retain many of the features of early vascular plants. Based on genome and transcriptome data, we identified *WUSCHEL-RELATED HOMEOBOX* (*WOX*) genes in *Selaginella kraussiana*, a model lycophyte that is convenient for *in vitro* culture and observations of organ formation and regeneration. *WOX* genes are key players controlling stem cells in plants. Our results showed that the *S. kraussiana* genome encodes at least eight members of the WOX family, which represent an early stage of WOX family evolution. Identification of *WOX* genes in *S. kraussiana* could be a useful tool for molecular studies on the function of stem cells in lycophytes.

## Introduction

Stem cells are characterized by their ability to self-renew in an undifferentiated state and their potential to differentiate into functional cells (Jaenisch and Young, 2008; Lander, 2009). All plant organs are derived from stem cells, and stem cells are also important in plant regeneration (Sugimoto et al., 2011; Xu and Huang, 2014). In angiosperms, stem cells are organized in a special environment; that is, the stem cell niche within the meristem (Scheres, 2007; Aichinger et al., 2012). Genes in the *WUSCHEL-RELATED HOMEOBOX* (*WOX*) family encode the key controllers of stem cell niche in many plant species. The WOX family homeobox proteins can be divided into three clades according to the time of their appearance during plant evolution: the ancient clade, the intermediate clade, and the WUS clade (Haecker et al., 2004; van der Graaff et al., 2009). The specification and organization of stem cells have become increasingly complex during the evolution of vascular plants, accompanied by increased complexity of *WOX* genes in diverse stem cell niches (Aichinger et al., 2012). To fully understand the complexities of stem cell activity, it would be useful to understand how stem cells functioned in the early evolution of vascular plants, as this may provide an evolutionary view of plant stem cells and stem cell niches.

**FIGURE 1:**
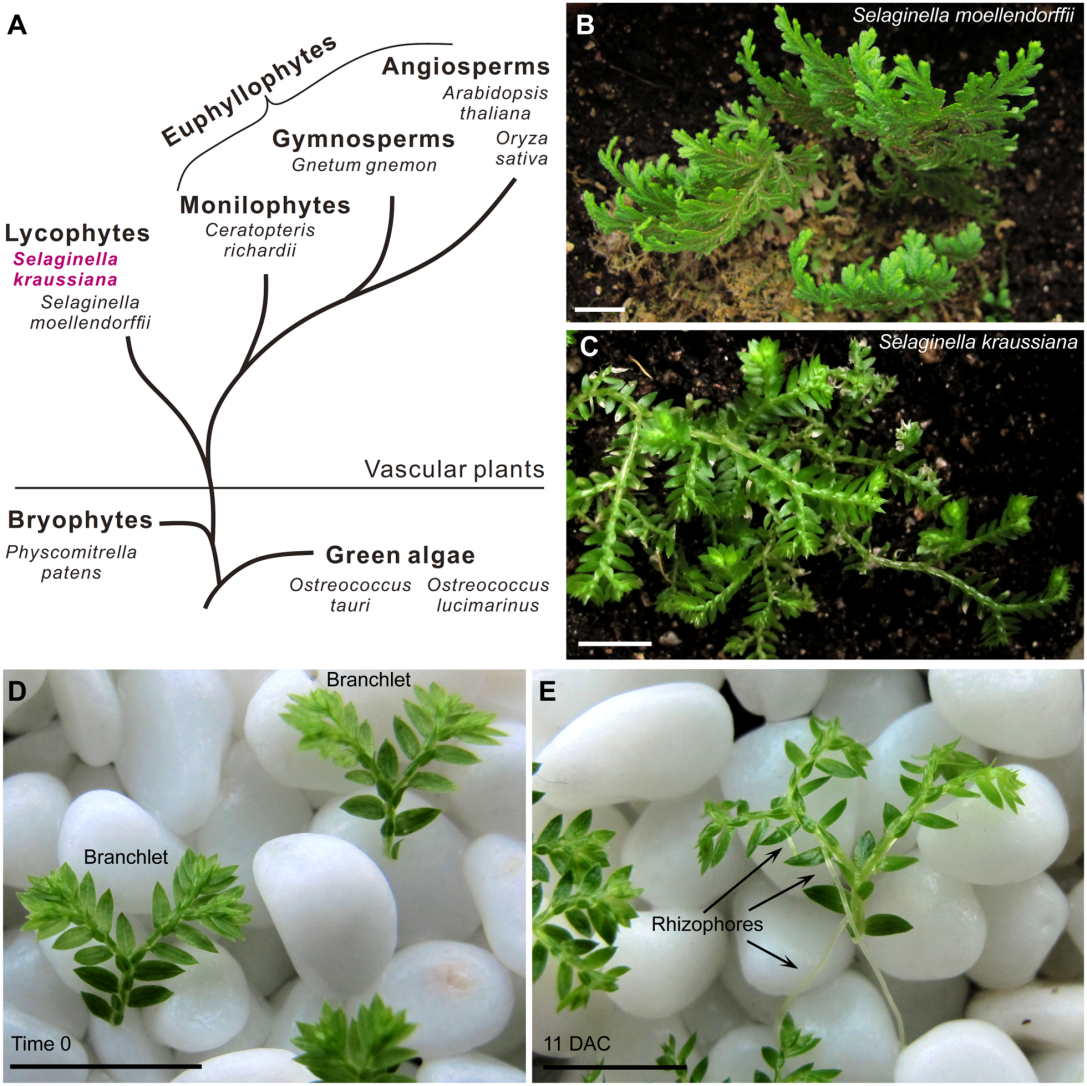
***Selaginella kraussiana* as a model plant for studies on lycophytes.** **(A)** Simplified evolutionary route of green plants, showing two existing branches of vascular plants; lycophytes and euphyllophytes. **(B,C)** Phenotypes of *S. moellendorffii* **(B)** and *S. kraussiana* **(C)**. Note that the stem of *S. moellendorffii* is upright **(B)**, while that of *S. kraussiana* is prostrate **(C)**. **(D,E)** *In vitro* culture system of *S. kraussiana*. Detached branchlets of *S. kraussiana* were cultured on wet stones at time 0 **(D)** and 11 DAC **(E)**. Growth of rhizophores that bear roots could be observed **(E)**. Scale bars, 1 cm in **(B–E)**.

More than 400 million years ago, the appearance of vascular plants was an important step in evolution during the colonization of land by plants (Bennici, 2007, 2008; Banks, 2009). Since then, vascular plants have diverged into several lineages, only two of which survive today; the lycophytes such as *Selaginella* (spikemosses), and the euphyllophytes consisting of monilophytes (ferns), gymnosperms, and angiosperms (**Figure 1A**; Raubeson and Jansen, 1992; Duff and Nickrent, 1999; Qiu and Palmer, 1999; Pryer et al., 2001; Qiu et al., 2007; Banks, 2009; Banks et al., 2011; Kenrick and Strullu-Derrien, 2014). Many lycophytes retain the typical features of early vascular plants, and therefore, are suitable for studies on the early evolution of vascular plants. For example, lycophytes have a simple and bifurcating apical meristem at the shoot and root tips (i.e., dichotomous branching), which is representative of early vascular plants during evolution (Banks, 2009). The genome of *Selaginella moellendorffii*, a model plant of lycophytes, was sequenced (Banks et al., 2011), and this greatly improves our knowledge on lycophytes. Identification of WOX family genes in *S. moellendorffii* suggests that lycophytes do not have the WUS clade (Deveaux et al., 2008; Mukherjee et al., 2009; van der Graaff et al., 2009).

*Selaginella kraussiana*, another model lycophyte, is easy to culture *in vitro* and suitable for studies on stem cells and regeneration. In this study, we identified *WOX* genes in *S. kraussiana* and analyzed their expression patterns in tissues based on genome and transcriptome data.

## Results

### *In Vitro* Culture System of *S. kraussiana*

*Selaginella* is a lycophyte lineage that separated from the euphyllophytes more than 400 million years ago (**Figure 1A**; Kenrick and Crane, 1997; Banks, 2009). Several species of *Selaginella* have been used to study different aspects of development. The stem phenotypes of *Selaginella* species are generally characterized by a prostrate or upright growth habit (Rost et al., 1997). *S. moellendorffii* has an upright stem (**Figure 1B**), and its genome has been sequenced (Banks et al., 2011). Different from *S. moellendorffii*, *S. kraussiana* is another commonly used species that has a typical prostrate stem (**Figure 1C**; Harrison et al., 2005; Floyd and Bowman, 2006; Prigge and Clark, 2006; Harrison et al., 2007; Otreba and Gola, 2011; Sanders and Langdale, 2013).

Compared with *S. moellendorffii*, *S. kraussiana* is more readily cultured *in vitro* and more amenable to morphological observations (Sanders and Langdale, 2013). In our conditions, the detached distal part of *S. kraussiana* seedlings with one or two branches grows readily on wet stones with water (**Figure 1D**). This growth method provides a humid environment that allows the survival of detached branchlets and also provides a physical support for stem, rhizophore, and root development. The rhizophore is a root-bearing organ in *Selaginella*. The detached *S. kraussiana* branchlets can grow continuously on the wet stones, producing more dichotomous shoot branches and rhizophores at the Y-shaped branch junctions (**Figure 1E**). In contrast, it is difficult to culture detached tissues of *S. moellendorffii in vitro*, and difficult to induce these tissues to form rhizophores.

### Organ Formation and Cell Fate Transition in *S. kraussiana*

**FIGURE 2:**
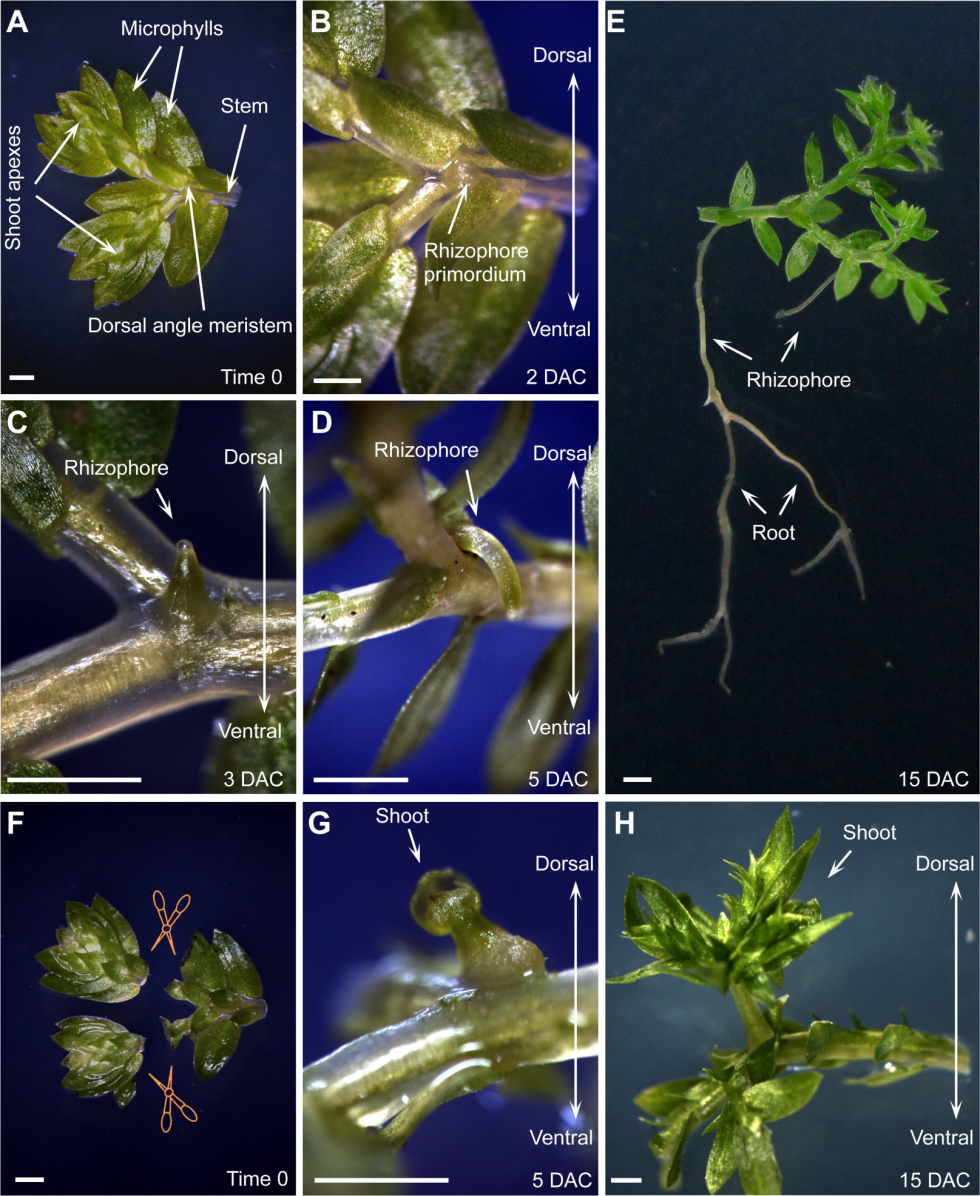
**Organ formation and regeneration in *S. kraussiana*.** **(A–D)** *In vitro* culture of *S. kraussiana*, showing rhizophore growth from detached branchlets at time 0 **(A)**, 2 DAC **(B)**, 3 DAC **(C)**, and 5 DAC **(D)**. Note that rhizophore primordium was observed from the dosal angle meristem at 2 DAC **(B)**. **(E)** Root formation from rhizophore. Note that the newly formed roots could bifurcate continuously. **(F–H)** Regeneration of shoot at angle meristem after excision of shoot apexes from branchlets. Shown are time-0 **(F)**, 5-DAC **(G)**, and 15-DAC **(H)** branchlets. Note that the regenerated shoot apex was observed from the dosal angle meristem at 5 DAC **(G)**. Detached branchlets were cultured on wet stones and removed to an agar plate to take pictures. Scale bars, 1 mm in **(A–H)**.

Using the *in vitro* culture system, *S. kraussiana* is suitable for studying organ formation and regeneration. The rooting process was clearly observed (**Figures 2A–E**; Otreba and Gola, 2011). In detached *S. kraussiana* branchlets, a rhizophore primordium was observed at the dorsal angle meristem located at the Y-shaped junction of dichotomous branching 2 days after culture (DAC; **Figures 2A,B**). Rhizophores continued to elongate (**Figures 2C,D**), and at 5 DAC the distal portion of the rhizophores started to bend (**Figure 2D**), indicating that their tips grew in response to gravity. The rhizophores continued to grow and produced bifurcating root tips (**Figure 2E**).

We also analyzed the regenerative ability of *S. kraussiana* in the *in vitro* culture system. It was reported that *Selaginella* has the ability to change the fate of angle meristem cells from rhizophore to shoot upon injury of the shoot apexes (Williams, 1937; Webster and Steeves, 1964; Webster, 1969; Wochok and Sussex, 1975). We repeated this experiment by excision of the two shoot apexes from the detached branchlet (**Figure 2F**). After excision, the new shoot apex regenerated within 5 DAC from the dorsal angle meristem (**Figure 2G**), where the rhizophore primordium usually grew in non-excised branchlets (**Figures 2B–D**). This suggests that the fate of stem cells within the angle meristem was changed from rhizophore or root to shoot during regeneration. The regenerated shoot apex grew continuously to form a seedling (**Figure 2H**). Overall, these observations confirmed that *S. kraussiana* is a good system for studying organ formation and regeneration.

### Identification of *WOX* Genes from Genome and Transcriptome Sequencing Data of *S. kraussiana*

**FIGURE 3:**
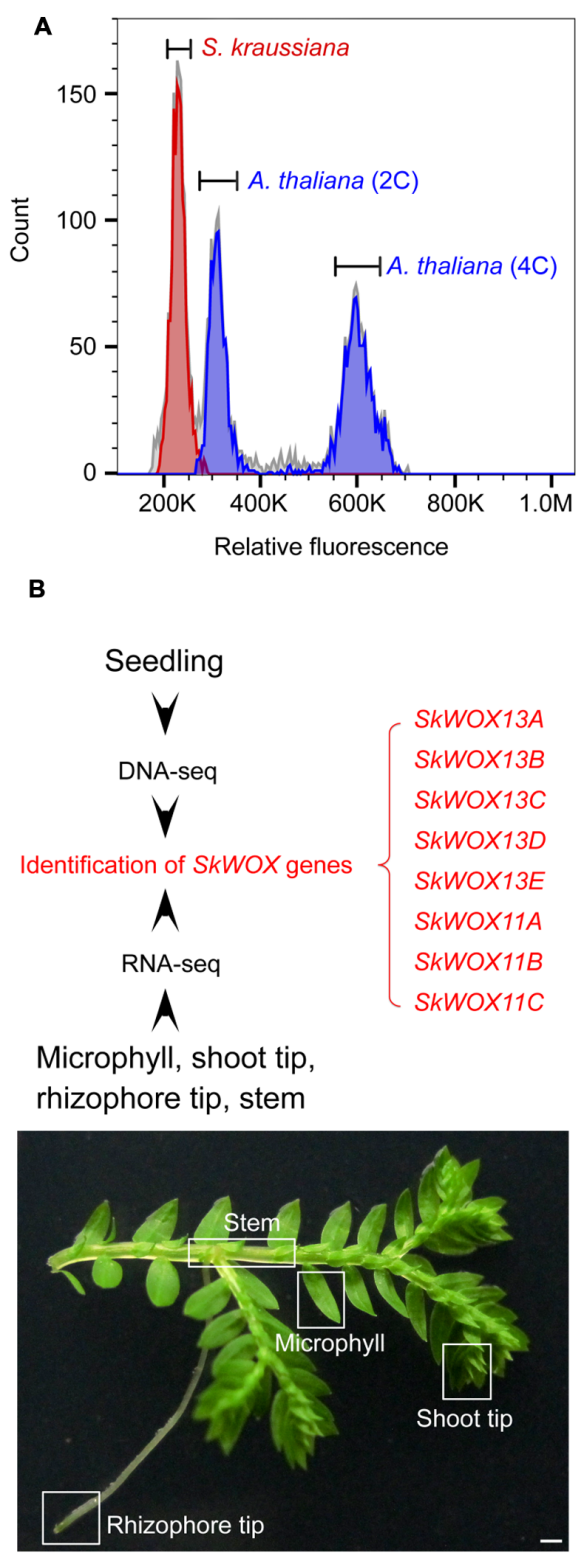
**Genome and transcriptome analyses of *S. kraussiana*.** **(A)** Flow cytometry analysis of genome size of *S. kraussiana*. Genome size of *S. kraussiana* (red peak, %CV = 4.74) was estimated using the internal reference standard *Arabidopsis thaliana* (indigo peaks: 2C, %CV = 4.81; 4C, %CV = 3.66). **(B)** DNA-seq and RNA-seq analyses of *S. kraussiana*. Genomic DNA from seedlings was used for DNA-seq and four different tissues were used for RNA-seq. Eight *SkWOX* genes (*SkWOX13A* to *E* and *SkWOX11A* to *C*) were identified.

To estimate the size of the *S. kraussiana* genome, we performed a flow cytometry analysis. The data showed that the genome of *S. kraussiana* is smaller than that of *Arabidopsis thaliana* (**Figure 3A**), consistent with the previous study (Little et al., 2007). We conducted a DNA-seq analysis of the *S. kraussiana* genome (**Figure 3B**). The draft assembly of contigs confirmed that the *S. kraussiana* genome is smaller than that of *A. thaliana* and similar to the length of the genome of *S. moellendorffii* (The Arabidopsis Genome Initiative, 2000; Banks et al., 2011).

To analyze the transcriptome of *S. kraussiana*, we performed an RNA-seq analysis using four different tissues: microphyll, shoot tip, rhizophore tip, and stem (**Figure 3B**). Since *WOX* genes are key regulators of stem cells in plants, we identified genes encoding members of the WOX family in *S. kraussiana* (*SkWOX* genes). Based on our DNA-seq and RNA-seq data, we identified eight genes predicted to encode a homeodomain similar to that of the WOX family proteins (**Figure 3B**; **Table 1**). We named the eight candidate *SkWOX* genes *SkWOX13A*–*E* and *SkWOX11A*–*C* according to the protein sequence similarity of their homeodomains to those of *A. thaliana* WOX (AtWOX) proteins. The cDNA or CDS sequences of the eight *SkWOX* genes were further confirmed by reverse transcription-polymerase chain reaction (RT-PCR) using total RNA from whole seedlings.

**Table 1:** Accession numbers of projects/sequences used in this study.

Projects/sequences	Deposition	Accession number
Whole Genome Shotgun project (DNA-seq, Draft assembly)^∗^	DDBJ/EMBL/GenBank	LDJE00000000
RNA-seq	GEO	GSE69388
BankIt1826187 skWOX11A	GenBank	KR870323
BankIt1826187 skWOX11B	GenBank	KR870324
BankIt1826187 skWOX11C	GenBank	KR870325
BankIt1826187 skWOX13A	GenBank	KR870326
BankIt1826187 skWOX13B	GenBank	KR870327
BankIt1826187 skWOX13C	GenBank	KR870328
BankIt1826187 skWOX13D	GenBank	KR870329
BankIt1826187 skWOX13E	GenBank	KR870330


*^∗^The version described in this paper is version LDJE01000000.*

**FIGURE 4:**
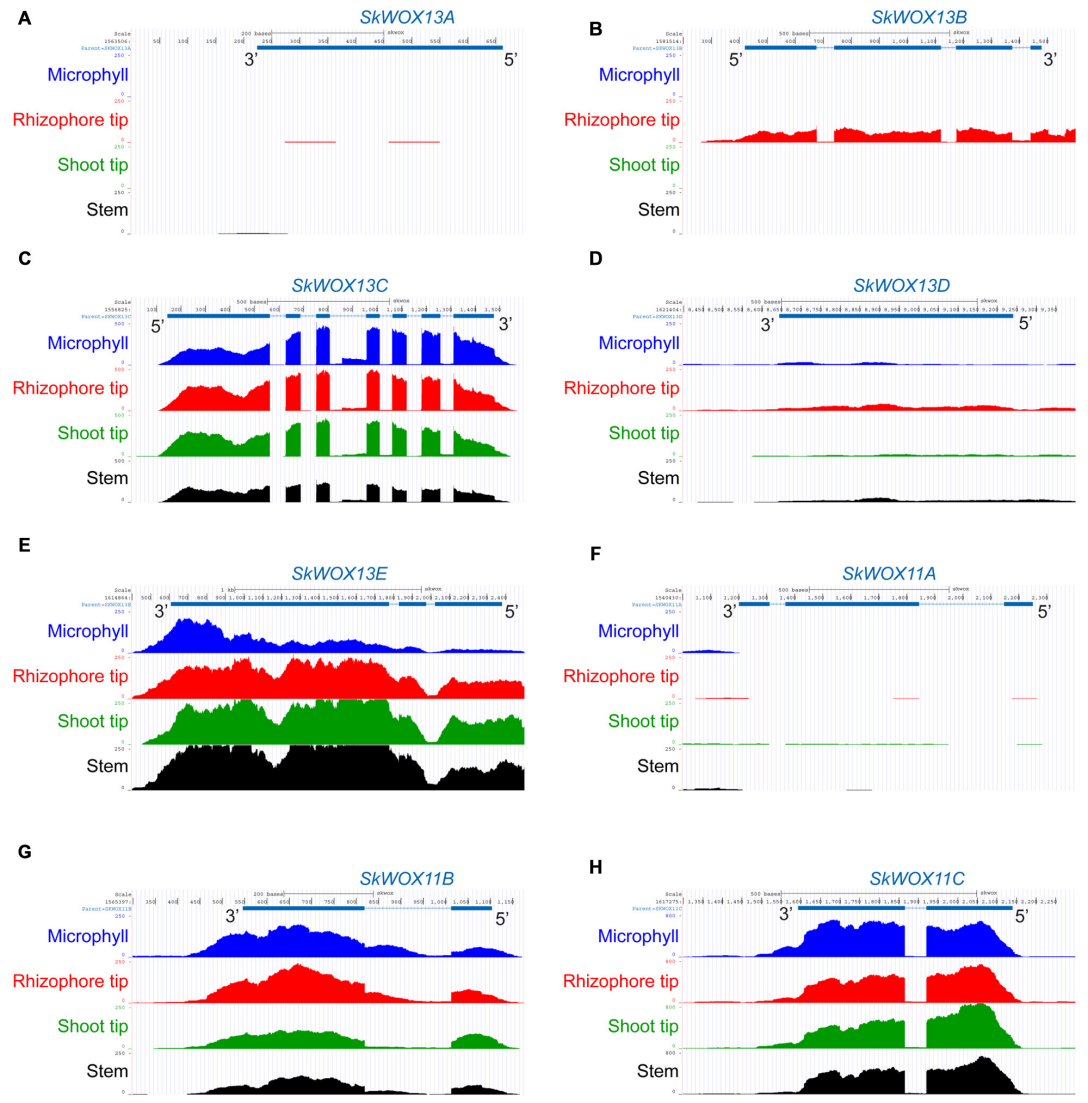
**Predicted expression profiles of *SkWOX* genes in *S. kraussiana.* (A–H)** RNA-seq analyses of expression patterns of *WOX* genes in *S. kraussiana*: *SkWOX13A* **(A)**, *SkWOX13B* **(B)**, *SkWOX13C* **(C)**, *SkWOX13D* **(D)**, *SkWOX13E* **(E)**, *SkWOX11A* **(F)**, *SkWOX11B* **(G)**, and *SkWOX11C* **(H)**. Peaks indicate RNA-seq reads aligned to the corresponding gene loci.

The predicted transcriptional profiles of *SkWOX* genes showed diverse patterns in the four tissues based on the RNA-seq data (**Figures 4A–H**). *SkWOX13A* and *SkWOX11A* were barely expressed in the tested tissues (**Figures 4A,F**), and there were low transcript levels of *SkWOX13D* in all tissues tested (**Figure 4D**). *SkWOX13C*, *SkWOX13E*, *SkWOX11B*, and *SkWOX11C* showed relatively high transcript levels in all four tissues (**Figures 4C,E,G,H**). Interestingly, *SkWOX13B* was preferentially expressed at the rhizophore tip (**Figure 4B**). These data suggested that each of the *SkWOX* genes may play specific role(s) in different tissues.

### Evolution of WOX Family Genes in Plants

**FIGURE 5:**
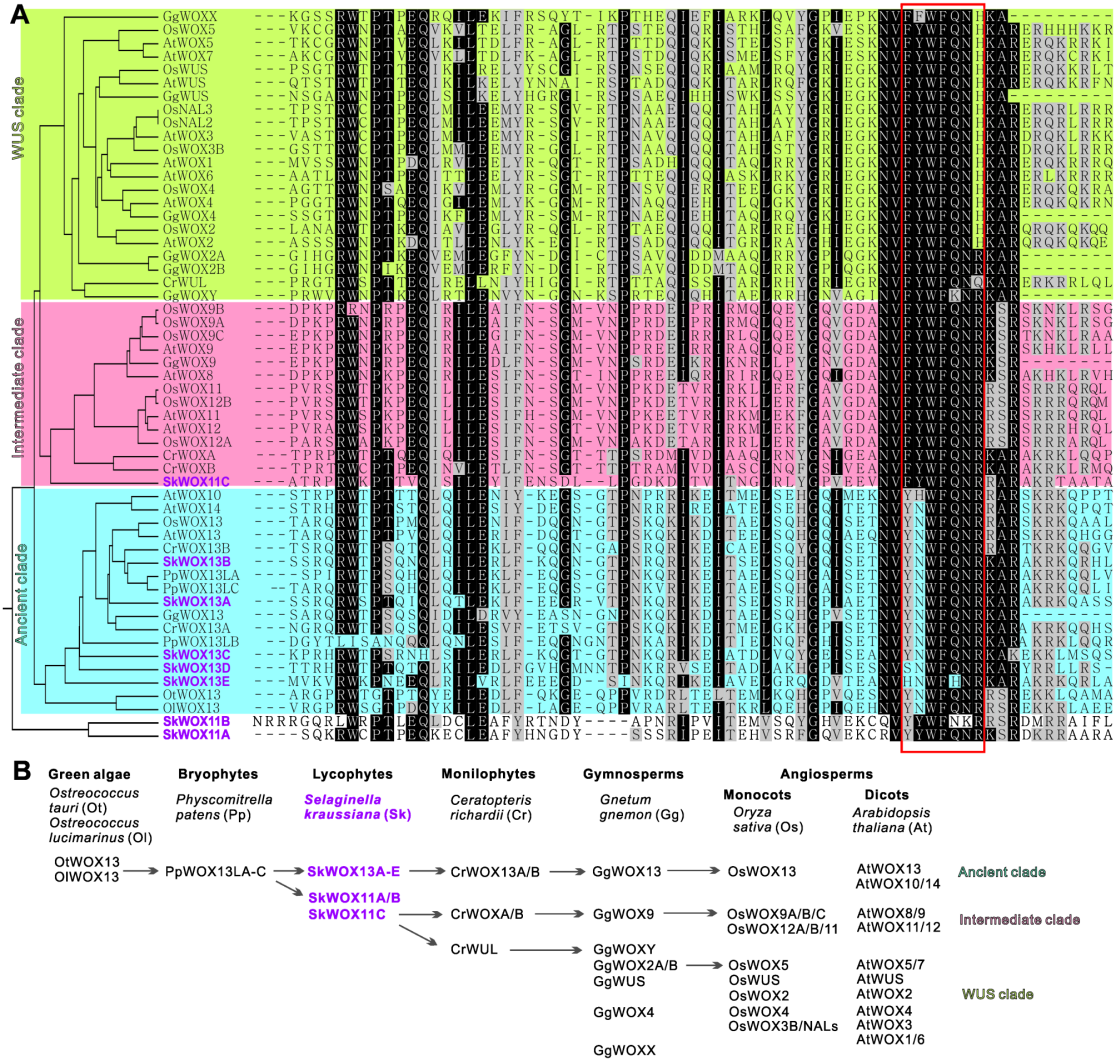
**Evolution of WOX family.** **(A)** Alignment of WOX homeodomains from different model plants. Phylogenetic analysis of homeodomain sequences was conducted using MEGA3.0 (Kumar et al., 2004). Protein sequences were obtained from published bioinformatics data (Haecker et al., 2004; Mukherjee et al., 2009; van der Graaff et al., 2009; Zhang et al., 2010; Nardmann and Werr, 2012, 2013; Lian et al., 2014; Zeng et al., 2015). Red box indicates sequence for classification of three clades. **(B)** Possible evolutionary route of WOX family genes.

To analyze the possible evolutionary history of the WOX family, we aligned the homeodomains of SkWOX proteins against the WOX homeodomains from the green algae *Ostreococcus tauri* (OtWOX13) and *Ostreococcus lucimarinus* (OlWOX13), the bryophyte/moss *Physcomitrella patens* (PpWOXs), the monilophyte/fern *Ceratopteris richardii* (CrWOXs), the gymnosperm *Gnetum gnemon* (GgWOXs), and the angiosperms *Oryza sativa* (OsWOXs) and *A. thaliana* (AtWOXs) (**Figure 5A**; Mukherjee et al., 2009; Nardmann and Werr, 2012, 2013; Sakakibara et al., 2014). Previous studies have suggested that particular peptide sequences in the WOX homeodomain can distinguish the three clades of WOX proteins (red box in **Figure 5A**): YNWFQNR for the ancient clade, FYWFQNR for the intermediate clade, and FYWFQNH for the WUS clade (Nardmann and Werr, 2012; Zeng et al., 2015).

Green algae and mosses only have the ancient clade of *WOX* genes, and moss *WOX* genes (*PpWOX13L*s) have been shown to play a role in apical stem cell formation and regeneration (Mukherjee et al., 2009; Sakakibara et al., 2014). Our data showed that *S. kraussiana* has at least five ancient-clade *WOX* genes, *SkWOX13A*–*E* (**Figures 5A,B**).

Consistent with previous genomic studies on *S. moellendorffii* (Mukherjee et al., 2009; van der Graaff et al., 2009; Lian et al., 2014), our data showed that the genome of *S. kraussiana* also encodes WOX proteins in the intermediate clade (**Figures 5A,B**). SkWOX11C has a FYWFQNR sequence in its homeodomain, typical of the intermediate clade. Interestingly, in *S. kraussiana* there is a lycophyte-specific clade of WOX proteins, SkWOX11A and B, which have a YYWFQNR (or YYWFNKR) sequence that appears to be transitional between the ancient (YNWFQNR) and the intermediate (FYWFQNR) clades (**Figures 5A,B**). Based on overall homeodomain sequence similarity, SkWOX11A/B separated from other WOX proteins in all the species tested in this study (**Figure 5A**), suggesting that this clade may have evolved far apart from the trunk road of the WOX family after they separated from SkWOX11C.

The *S. kraussiana* genome does not encode WUS-clade genes. The fern *C. richardii* contains WOX members similar to WUS-clade proteins, such as CrWUL (Nardmann and Werr, 2012). Sequence analysis showed that the CrWUL protein from *C. richardii* and GgWOXY and GgWOX2A/B from the gymnosperm *G. gnemon* show high sequence similarity to WUS-clade proteins (**Figure 5A**). However, none of these proteins contain the WUS-clade sequence FYWFQNH in the homeodomain (**Figure 5A**; Mukherjee et al., 2009; Nardmann and Werr, 2012, 2013; Zeng et al., 2015). Instead of FYWFQNH, GgWOXY and GgWOX2A/B have an intermediate sequence FYWFQNR (**Figure 5A**). This suggests that CrWUL, GgWOXY, and GgWOX2A/B might represent a transitional evolutionary stage from intermediate to WUS-clade proteins, although these proteins are usually included in the WUS clade (**Figures 5A,B**; Mukherjee et al., 2009; Nardmann and Werr, 2012, 2013; Zeng et al., 2015). The WUS-clade genes have further evolved in gymnosperms and angiosperms (**Figures 5A,B**) (Mukherjee et al., 2009; Nardmann et al., 2009; van der Graaff et al., 2009; Lian et al., 2014).

## Discussion

Lycophytes retain many features of vascular plants at the early stage of landing, and therefore, are useful for understanding the early evolution of vascular plants (Banks, 2009). *S. moellendorffii* and *S. kraussiana* are two commonly used lycophyte model plants that differ according to their stem phenotype (typical upright and prostrate stems, respectively). Both of them have merits for studies on lycophytes. Compared with *S. moellendorffii*, *S. kraussiana* has several advantages for studying organ formation and stem cell functions. First, *S. kraussiana* grows rapidly and is easy to culture *in vitro*. Second, it is easy to trace rhizophore and root development in *S. kraussiana*. Third, it is easy to observe the fate transition of stem cells during organ regeneration in *S. kraussiana*.

The genome sequence of *S. moellendorffii* has been reported previously (Banks et al., 2011). This genomic information has greatly improved our understanding of the evolution of the plant kingdom. In this study, we provided raw data of DNA-seq of the *S. kraussiana* genome and RNA-seq of four different *S. kraussiana* tissues (**Table 1**). These sequencing data provide a useful tool to study the developmental regulation of *S. kraussiana* using a molecular approach.

The *WOX* genes have evolved from ancient to intermediate to WUS clades, accompanied by the evolution of stem cell function in plants (Haecker et al., 2004; Mukherjee et al., 2009; van der Graaff et al., 2009; Aichinger et al., 2012; Lian et al., 2014; Zeng et al., 2015). The *WOX* family could be a useful tool for studying stem cells in plants. Green algae and mosses only have the ancient clade. Our data from *S. kraussiana* and previous studies in *S. moellendorffii* (Deveaux et al., 2008; Mukherjee et al., 2009; van der Graaff et al., 2009) showed that lycophytes have ancient and intermediate clades. Ferns, gymnosperms and angiosperms have all the three clades. Therefore, the intermediate clade might first originate in the common ancestor of lycophytes and euphyllophytes, suggesting that this clade evolved specifically in vascular plants. Conversely, there are no WUS-clade members in lycophytes, suggesting that stem cells in lycophytes are still at an early evolutionary stage.

In this study, we identified a lycophyte-specific clade in *S. kraussiana*; the evolutionary position of this clade is probably between the ancient and intermediate clades. This clade may have separated from the intermediate clade and further evolved in lycophytes. Studies on the functions of *S. kraussiana WOX* genes, together with studies on stem cell activities, organ formation, and regeneration in lycophytes using a molecular approach, may help us to understand the developmental mechanisms in the early evolution of vascular plants.

## Materials and Methods

### Plant Materials, Culture Conditions, and Microscopy

Plants of *S. kraussiana* were grown at 26°C under a 16-h light (∼5000 Lux)/8-h dark photoperiod in a greenhouse or plant chamber. Microscopy analyses were performed using a Nikon SMZ1500 microscope (Nikon, Tokyo, Japan).

### Genome Sequencing and Assembly

We extracted genomic DNA from seedlings of *S. kraussiana* and then employed whole-genome shotgun strategy to decode the genome of *S. kraussiana*. DNA library construction and deep sequencing were performed by Genergy Biotechnology Co. Ltd. (Shanghai, China). The average length of the inserted library was about 200 bp. The paired-end library was sequenced by Illumina HiSeq 2000 platform following the manufacturer’s instructions (Illumina Inc., San Diego, CA, USA.). About 289 millions (289,347,796) of pair-ended reads were generated, which corresponded to 250× sequencing depth. The pair-ended reads were assembled with ABySS software v1.5.1 (Simpson et al., 2009), using the default parameters in ABySS assembler. A total of 49,647 contigs were larger than 500 bp and 8561 contigs were larger than N50 (3096 bp).

### RNA-seq and Alignment

We extracted RNA samples from four different tissues of *S. kraussiana*: microphyll, shoot tip, rhizophore tip, and stem. The RNA extracts were used to construct RNA libraries and sequenced by Genergy Biotechnology Co. Ltd. (Shanghai, China). The average length of inserted library was about 300 bp. The pair-ended libraries were sequenced by Illumina HiSeq 2000. A total of 936843033 (stem, 208693928; leaf, 240658608; rhizophore, 229142616; seeding, 258347936) pair-ended reads were obtained. The first ten base pairs were trimmed off the reads. The trimmed reads were aligned against the assembled genome sequence using TopHat V2.0 and then analyzed by CuffLinks V2.1 (Trapnell et al., 2010).

### Genome Annotation by MAKER

To identify *SkWOX* genes, we used MAKER annotation pipeline (v 2.31) to annotate the assembled genome of *S. kraussiana*. The aligned RNA-seq dataset were used as EST evidences. The protein sequences of related *S. moellendorffii* species were used as protein evidences. We trained SNAP predictor with the protein sequences of *S. moellendorffii*. The repeat sequences dataset from *S. moellendorffii* were used as the input of RepeatMasker (v 4.0.5). After annotated by MAKER, those genes which were not supported by evidence or did not have a PFAM domain were filtered out. The genes were annotated by InterProScan (version: 5.15-54.0) (Jones et al., 2014).

### Flow Cytometry

Flow cytometry analyses to determine the nuclear DNA contents of *S. kraussiana* and *A. thaliana* were performed according to the method described previously (Galbraith et al., 1983; Dolezel et al., 2007; Little et al., 2007). Approximately 150-mg rosette leaves of 3-week-old *A. thaliana* (Col-0) plants and 60-mg young branchlets of *S. kraussiana* were homogenized on ice in 1-ml ice-cold modified Galbraith’s buffer (45-mM MgCl_2_, 30-mM sodium citrate, 20-mM MOPS, 1% (vol/vol) Triton X-100, pH7.0, 5-mM sodium metabi-sulfite and 5-μl β-mercaptoethanol) complemented with 50-μg/ml PI and 100-μg/ml RNase. The homogenate was filtered through a 40-μm nylon cell strainer (Falcon, BD Biosciences, San Jose, CA, USA), then the filtrate with an additional 50-μg RNase was incubated at 37°C for 30 min. Samples were kept on ice in the dark until analysis. Approximately 5000 particles were measured at a low sample flow rate using a CytoFLEX flow cytometer (585/42 BP channel, 488-nm laser, Beckman Coulter Inc., Brea, CA, USA), and the data were analyzed with FlowJo_V10 software.

### RT-PCR

RNA was extracted from seedlings or rhizophores of *S. kraussiana* using TRIzol Reagent (Life Technologies, Carlsbad, CA, USA) and used as the template for reverse transcription as previously described (Xu et al., 2003; He et al., 2012). The RT-PCR analyses were performed using the following gene-specific primers:

5′-GTAAGTAAGCTTTTCAGATG-3′ and 5′-GTATGTGATCTATAAGCTTG-3′ for *SkWOX13A*; 5′-CACATGCACCTTATATTCCTCC-3′ and 5′-CGTACATACCATTGCATGAG-3′ for *SkWOX13B*; 5′-GCAGAACGAGGAGATTAGCG-3′ and 5′-GTCGTGTTAGCTCTAGTATAG-3′ for *SkWOX13C*; 5′-CAATCGCAACGTACGTTACAG-3′ and 5′-GTACTTTGTCGAGAAGGACAC-3′ for *SkWOX13D*; 5′-TAGGATCAAGTGACCACCTG-3′ and 5′-CAACTCAGAAGTCTGATGATC-3′ for *SkWOX13E*; 5′-CGAGTCTCTCACACTCAGAC-3′ and 5′-GAGCCTGAACCTGAACACAG-3′ for *SkWOX11A*; 5′-GTCTCCGTGAAGAAGCCCAAAG-3′ and 5′-GCGCACACAGCGGTGCACTG-3′ for *SkWOX11B*; and 5′-GGTTCGTGAGTCATTTGTGA-3′ and 5′-GTCGCCAGACTTACGAATTC-3′ for *SkWOX11C*.

## Accession Numbers

The Whole Genome Shotgun project (DNA-seq data) has been deposited in DDBJ/EMBL/GenBank. RNA-seq data have been deposited at GEO. The sequences of *WOX* genes have been deposited in GenBank. Accession numbers are listed in **Table 1.**

## Author Contributions

All authors listed, have made substantial, direct and intellectual contribution to the work, and approved it for publication.

## Conflict of Interest Statement

The authors declare that the research was conducted in the absence of any commercial or financial relationships that could be construed as a potential conflict of interest.
